# Hepatitis C elimination in Sweden: Progress, challenges and opportunities for growth in the time of COVID‐19

**DOI:** 10.1111/liv.14978

**Published:** 2021-06-30

**Authors:** Sarah Blach, Marianne Blomé, Ann‐Sofi Duberg, Anna Jerkeman, Martin Kåberg, Per‐Erik Klasa, Martin Lagging, Devin Razavi‐Shearer, Homie Razavi, Soo Aleman

**Affiliations:** ^1^ Department of Epidemiology Center for Disease Analysis Foundation Lafayette CO USA; ^2^ Faculty of Medicine Department of Translational Medicine Clinical Infection Medicine Lund University Malmö Sweden; ^3^ Faculty of Medicine and Health Department of Infectious Diseases Örebro University Örebro Sweden; ^4^ Stockholm Needle Exchange Stockholm Centre for Dependency Disorders Stockholm Sweden; ^5^ Division of Infection and Dermatology Department of Medicine Huddinge Karolinska Institute Karolinska University Hospital Huddinge Stockholm Sweden; ^6^ Addiction Medicine PRIMA Maria OST Clinic Stockholm Sweden; ^7^ Department of Infectious Diseases/Virology Institute of Biomedicine Sahlgrenska Academy University of Gothenburg Gothenburg Sweden; ^8^ Department of Clinical Microbiology Region Västra Götaland Sahlgrenska University Hospital Gothenburg Sweden; ^9^ Department of Medicine Huddinge Karolinska Institute Stockholm Sweden; ^10^ Department of Infectious Diseases Karolinska University Hospital Stockholm Sweden

**Keywords:** COVID‐19, elimination, hepatitis C virus, Sweden

## Abstract

**Background & Aims:**

In 2014, the burden of hepatitis C virus (HCV) in Sweden was evaluated, to establish a baseline and inform public health interventions. Considering the changing landscape of HCV treatment, prevention, and care, and in light of the COVID‐19 pandemic, this analysis seeks to evaluate Sweden's progress towards the World Health Organization (WHO) elimination targets and identify remaining barriers.

**Methods:**

The data used for modelling HCV transmission and disease burden in Sweden were obtained through literature review, unpublished sources and expert input. A dynamic Markov model was employed to forecast population sizes and incidence of HCV through 2030. Two scenarios (‘2019 Base’ and ‘WHO Targets’) were developed to evaluate Sweden's progress towards HCV elimination.

**Results:**

At the beginning of 2019, there were 29 700 (95% uncertainty interval: 19 300‐33 700) viremic infections in Sweden. Under the base scenario, Sweden would achieve and exceed the WHO targets for diagnosis, treatment and liver‐related death. However, new infections would decrease by less than 10%, relative to 2015. Achieving all WHO targets by 2030 would require (i) expanding harm reduction programmes to reach more than 90% of people who inject drugs (PWID) and (ii) treating 90% of HCV + PWID engaged in harm reduction programmes and ≥7% of PWID not involved in harm reduction programmes, annually by 2025.

**Conclusions:**

It is of utmost importance that Sweden, and all countries, find sustainability in HCV programmes by broadening the setting and base of providers to provide stability and continuity of care during turbulent times.

**Abbreviations**
COVID‐19: coronavirus disease 2019
DAA: direct acting antiviral treatment
EMCDDA: European Monitoring Centre for Drugs and Drug Addiction
HCV: hepatitis C virus
NSP: needle and syringe programme
OST: opiate substitution therapy
PWID: people who inject drugs

**Lay Summary**

Sweden is on‐track to achieve three of the four World Health Organization targets for hepatitis C virus (HCV) elimination, but reducing new infections will require increased access to HCV treatment for people who inject drugs. COVID‐19 exposed weaknesses in HCV outreach and care programmes that could be addressed by expanding the base of providers who can diagnose and treat HCV. This would alleviate the burden on infectious disease clinics and hospitals dealing with COVID‐19, while ensuring better continuity of care for HCV patients.

## 1 BACKGROUND

In 2014, the burden of hepatitis C virus (HCV) in Sweden was evaluated and published, to establish a baseline and inform public health interventions.^1^ Just 2 years later, the 69th World Health Assembly unanimously voted to adopt a Global Health Sector Strategy for the elimination of hepatitis as a public health threat by 2030.^2^ Those targets include increasing the proportion of diagnosed patients to 90%, increasing treatment coverage to 80%, reducing new infections by 90% and reducing mortality by 65%.^2^

Since that initial analysis in 2014, Sweden has made large strides in HCV treatment and prevention. Access to HCV treatments has been established free of charge to patients through cost sharing agreements with the Swedish states and healthcare regions (70% of costs covered by the state and 30% covered by the healthcare region). National treatment guidelines have been expanded to include people who inject drugs (PWID) and those with acute infections. Additionally, harm reduction programmes have been established across the country and strengthened to include HCV treatment and care.^3^, ^4^ In Stockholm, harm reduction programmes have been effective in lowering the viremic prevalence of HCV among participants from 55% to 40%.^5^

The emergence of the severe acute respiratory syndrome coronavirus 2 (SARS‐CoV‐2), which causes coronavirus disease 2019 (COVID‐19), resulted in a unique challenge for healthcare systems globally and in Sweden. As of 18 November 2020, 196 446 people in Sweden tested positive for COVID‐19 with 6321 total deaths reported.^6^ Although Sweden never experienced a ‘hard lockdown’, COVID‐19 impacted HCV care as well as harm reduction and addiction treatment services. Experiences vary by region and clinic and range from disrupted outreach activities (Stockholm) to a temporary pause in treatment starts (Örebro and Skåne) (personal communication with the authors). In Gothenburg, for example, the infectious disease clinic was used as the COVID‐19 clinic, resulting in a temporary discontinuation, from April until May 2020, in treatment within that setting.

At a national level, the numbers of patients diagnosed with HCV (as reported to Folkhälsomyndigheten [The Public Health Agency of Sweden]) were reduced by 27% from January to October 2020, compared with the same time frame in 2019.^7^ Similarly, HCV treatment starts were reduced by 55%, from January to October 2020, compared with the same time frame in 2019 (personal communication with Ola Weiland, InfCare Hepatitis Register).^8^ Some reduction in treated patients could be attributed to a waning population of warehoused patients; however, a decrease in newly diagnosed patients and restricted availability to outreach programmes was also experienced (either due to not yet started activities or ongoing activities restricted due to COVID‐19). A further concern is that independently of COVID‐19, Sweden has experienced large loss to follow up among diagnosed HCV infected patients in care at hospitals with available treatment, identifying an unmet need to expand care beyond this setting ^9^

Considering the changing landscape of HCV treatment, prevention and care in Sweden, and in light of the COVID‐19 pandemic, this analysis seeks to evaluate Sweden's progress towards the World Health Organization (WHO) elimination targets and identify remaining barriers.

## 2 METHODS

The data used for modelling were obtained through literature review, unpublished sources and expert input. Next, a dynamic Markov model was used to forecast the disease burden and transmission of HCV. The methodology for calculating new infections due to transmission among PWID is described in the Appendix. Inputs are included in the Appendix and briefly described here.

### 2.1 Hepatitis C virus disease burden model

Hepatitis C virus prevalence, prevalence by age and sex, and the number of persons previously diagnosed and treated were used to seed and calibrate the model. Many of these inputs have been described in detail previously, with newer inputs described briefly here.^1^ The annual number of cases notified to the Swedish Public Health Authority was used to estimate the number of persons newly diagnosed. Beginning in 2020, the basis for notification changed from anti‐HCV to HCV‐RNA or HCV‐Ag, and labs introduced reflex testing for anti‐HCV positive samples. To estimate the number of newly diagnosed viremic cases, data reported prior to 2020 were adjusted assuming a viremic rate of 77%.^7^, ^10^, ^11^, ^12^ The number of patients initiated on direct acting antiviral (DAA) treatment per year and the total number of unique patients treated from 2014 to 2019 were accessed through the Prescription Register, National Board of Health and Welfare. Summing annual treated patients resulted in a total that was ~12% higher than the total unique patients treated, so the annual estimates were adjusted down to account for possible double counting of patients initiating and completing treatment in different years. An Sustained virological response (SVR) of 95% was considered from 2014 to 2016, increasing to 97% after 2017. The number of patients transplanted who were positive for anti‐HCV from 1990 to 2018 was provided by Scandiatransplant (Scandiatransplant, 2019 data, requested by Dr Aleman).

### 2.2 Subpopulations and the hepatitis C virus transmission module

To model transmission of HCV among PWID, data around population size, harm reduction and injecting behaviours were collected from published and unpublished sources and were informed by expert consensus if empirical data were unavailable. PWID was defined as persons who have injected drugs within the last year, with stimulants (59%; primarily amphetamine) and opioids (34%; primarily heroin) as the most frequently injected drugs in Sweden (National InfCare Needle Syringe Program, 2019 data, Dr Kåberg). Participation data for opiate substitution therapy (OST) and needle and syringe programmes (NSP) were available through the InfCare NSP quality register and from data reported to the European Monitoring Centre for Drugs and Drug Addiction (EMCDDA). Assumptions around injecting behaviour were available from published sources or estimated using expert consensus. A full description of the inputs identified for transmission modelling, as well as the value in the year of report, can be found in the Appendix, Section 1.

Additionally, the size of the HCV‐infected population in Swedish prisons was estimated using data on the number of prisoners, annual turnover of prisoners and the proportion testing positive for anti‐HCV.

### 2.3 Modelled scenarios for the elimination of hepatitis C virus in Sweden

Once the model was developed, a variety of ‘what‐if’ scenarios were run to evaluate the impact of future decisions. These scenarios were first envisaged before the COVID‐19 pandemic began, but a new scenario has been added to evaluate the impact of real‐world delays in treatment that have been experienced in Sweden as a result of the pandemic and is discussed in more detail in the next section. The scenarios included are as follows.

A baseline scenario (Base 2019) was developed using empirical diagnosis and treatment data through 2019. After 2019, screening was assumed to remain constant, resulting in fewer newly diagnosed cases each year (Table 1). Assuming no major improvements in case finding or linkage to treatment or harm reduction, the number of patients starting treatment each year would decrease (Table). However, as treatment in harm reduction becomes commonplace, the proportion of treatments directed to NSP and OST clients was assumed to increase (assuming a constant proportion of clients on NSP and OST are initiated on treatment each year). This assumes that outreach programmes from hospital settings would be improved substantially, or that prescription/treatment by healthcare providers outside of hospital settings (usually from department of infectious diseases in Sweden) would be routine practice.

**TABLE 1:** Annual number diagnosed and initiating treatment, as well as treatment eligibility and SVR under the 2019 base and WHO targets scenarios, 2018‐2030

Scenario input	Scenario	2018	2019	2020	2021‐2022	2023‐2024	2025‐2030	Cumulative 2019‐2030
Newly diagnosed (viremic)	All scenarios	1200	1100	1000	1000	970	950	11 800
Initiating treatment								
General population	2019 Base	5300	4200	2700	2700	2100	2100	19 100
	WHO Targets	5300	4200	2200	1800	700	800	9200
PWID and prisoners	2019 Base	520	610	500	460	430	390	5100
	WHO Targets	520	610	500	1400	1800	1700	15 100
Treatment eligibility, fibrosis stage	All scenarios	≥F0	≥F0	≥F0	≥F0	≥F0	≥F0	—
Treatment eligibility, age (y)	All scenarios	20‐74	20‐74	20‐74	20‐74	20‐74	20‐74	—
SVR	All scenarios	97%	97%	97%	97%	97%	97%	—

Abbreviations: PWID, people who inject drugs; WHO, World Health Organization.

Additionally, a scenario was developed to identify the steps needed to achieve the WHO elimination targets (90% diagnosed, 80% treated, 65% reduction in mortality and 90% reduction in incidence) by 2030 (WHO 2030). This scenario considered increased diagnosis and treatment both in the general population and among PWID. With the PWID population, the impact of increased uptake of harm reduction services was also calculated (Table 1).

### 2.4 Uncertainty analysis and the impact of COVID‐19

Crystal Ball release 11.1.2.3.500 was used to calculate uncertainty intervals (UIs) and conduct sensitivity analyses. β‐PERT distributions were used for all uncertain inputs. A Monte Carlo simulation with 1000 trials was used to estimate 95% UIs.

To evaluate the impact of COVID‐19 on elimination efforts, the number of patients initiated on treatment per month in 2019 and 2020 were accessed through the IQVIA prescription claims database (IQVIA, formerly QuintilesIMS, is a large aggregator of pharmacy and prescription data). Projections based on monthly diagnosis and treatment data from the first half of 2020 were used to estimate the number of patients treated in 2020. Because the full extent of disruptions due to COVID‐19 are yet to be seen, a variety of sensitivity analyses were explored and compared assuming changing treatment levels over the next 5 years.

To examine the impact of faster scale‐ups in harm reduction services, three scenarios achieving 90% of PWID engaged in NSP by 2023 were also developed, as follows: (i) 90% of PWID engaged in NSP by 2023, no treatment; (ii) 90% of PWID engaged in NSP by 2023, treatment at base levels; and (iii) 90% of PWID engaged in NSP by 2023, WHO Targets.

## 3 RESULTS

**FIGURE 1:**
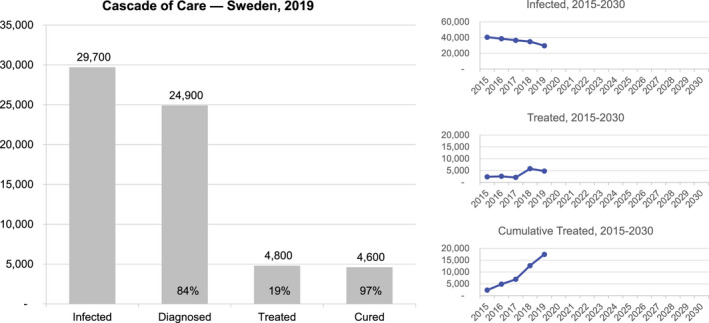
Cascade of care, 2019

**FIGURE 2:**
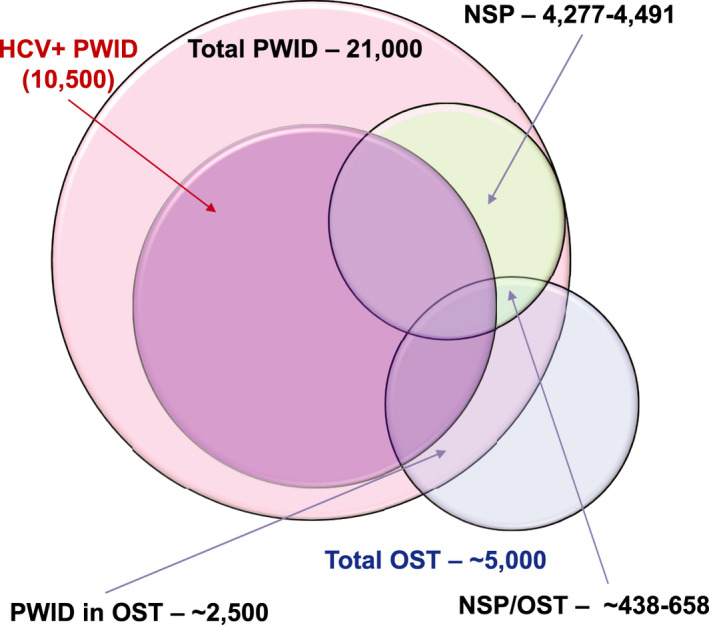
Size of the OST, NSP and PWID populations, 2019. NSP, needle and syringe programs; OST, opiate substitution therapy; PWID, people who inject drugs

At the beginning of 2019, there were an estimated 29,700 (95% UI: 19 300‐33 700) viremic infections in Sweden (0.30% [95% UI: 0.19%‐0.34%] HCV‐RNA+) (Table 2). Of these, more than 80% had already been diagnosed; however, due to incomplete linkage to care and loss to follow‐up, fewer than 20% of prevalent infections were initiated on treatment in the same year (Figure 1). There were an estimated 21 000 PWID in Sweden, more than half of whom primarily injected amphetamines. Approximately, 23% of PWID were engaged in harm reduction (NSP, OST or both) programmes, and 10 500 of all PWID were HCV‐RNA+ (Figure 2). Within harm reduction programmes, an estimated 15% of NSP participants and 26% of PWID on OST were initiated on HCV treatment annually, or approximately 540 PWID treated in 2018. In 2019, the model calculates that there would be 1200 new HCV infections (including reinfection) occurring annually (incidence rate 28.7/100). Because the risk of transmitting HCV decreases among PWID engaged in harm reduction services, the majority of new infections (>80%) was estimated to occur among PWID who were not in contact with harm reduction services.

Before considering the impact of COVID‐19, 1070 persons were estimated to be newly diagnosed with viremic HCV in Sweden with 3200 initiated on treatment in 2020. These estimates would represent a consistent number of annually diagnosed new infections and a decrease in treated patients from 4800 in 2019. However, data from the first 10 months of 2020 suggest that the actual number of new viremic diagnoses will be closer to 778, a 27% reduction from 2019 (Appendix, Section 2). Similarly, InfCare Hepatitis data suggest that only 2110 patients would be forecast to receive HCV treatment in 2020, a 56% reduction relative to the number of patients treated in 2019, and 34% lower than initial (pre‐COVID‐19) forecasts for 2020 (Appendix, Section 2).

**FIGURE 3:**
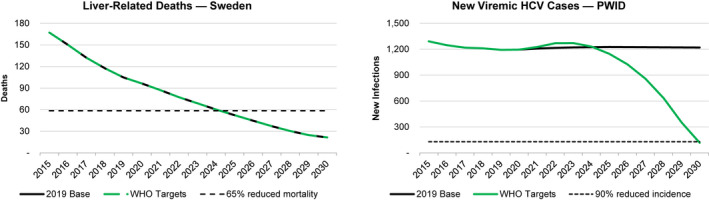
Progress towards the WHO 2030 targets for liver‐related deaths and incidence of HCV under the Base 2019 and WHO Targets scenarios, 2015‐2030, based on the assumptions provided in Table 1. *Liver related deaths for the 2019 Base and WHO targets overlap. HCV, hepatitis C virus; WHO, World Health Organization

Under the 2019 base scenario, Sweden was projected to achieve and exceed the WHO targets for diagnosis, treatment and liver‐related deaths (Figure 3, Table 2). Current efforts to treat HCV among PWID in OST and NSP would reduce total prevalent infections; however, new infections were projected to decrease by less than 10% relative to a 2015 comparison point (Figure 3). Under this scenario, 9700 people would be newly diagnosed, and 24 200 initiated on treatment between 2021 and 2030, leading to an 87% reduction in liver related deaths by 2030.

Considering the impact of COVID‐19 in 2020, and assuming the number of patients diagnosed and initiated on treatment returned to previously forecasted levels by 2021, Sweden would still achieve the targets for diagnosis, treatment and mortality, but would not achieve the target for incidence. However, compared with the 2019 base scenario, there would be an incremental 31 cases of HCC and 40 liver‐related deaths by 2030. If disruptions continued for 2 or 5 years, further increases in excess HCC cases (74 and 93, respectively) and liver related deaths (94 and 118, respectively) could be expected (Appendix, Section 3).

**FIGURE 4:**
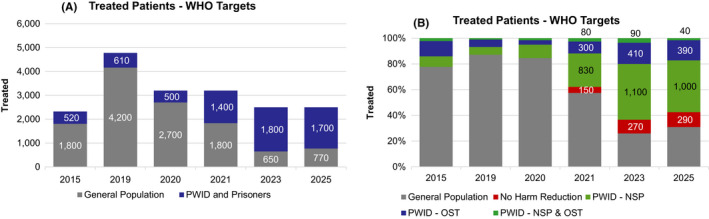
Distribution of treated patients under the WHO 2030 target scenario: (A) absolute number treated in the general population and PWID and prisoner population; (B) percent of total treatments, by population and harm reduction status. PWID, people who inject drugs; WHO, World Health Organization

Achieving all of the WHO targets by 2030 would require increased efforts to sustain and expand treatment in hard‐to‐reach populations. Outreach programmes with easier access to HCV treatment, with expansion of HCV treatment beyond hospital‐settings would be needed to sustain the treatment rate. Another option would be to broaden the provider base with prescription of HCV drugs of physicians in NSP, OST, primary care and so on. Harm reduction programmes would need to expand to cover at least 80% of PWID. Although this could be achieved through a variety of scenarios, the scenario modelled here included a 12% annual increase in participants accessing NSP programmes and a 10% annual increase in participants accessing OST, beginning in 2020, to reach ≥90% coverage by 2030. Next, 90% of PWID engaged in harm reduction settings would need to be initiated on HCV treatment annually by 2025. This could be achieved through linear increases in the proportion of patients treated annually beginning in 2021 (Figure 4). Finally, linear increases in the proportion of PWID treated for HCV outside of harm reduction settings would need to reach 7% annually (maximum 290 patients annually) by 2025 (Figure 4).

Increasing PWID engagement in NSP programmes to reach 90% by 2023 could reduce the number of new infections among PWID by 27%, even in the absence of treatment (Appendix, Section 4). When combined with base treatment levels, incidence could be reduced by 55%. The WHO targets could be achieved with 90% of PWID engaged in NSP by 2023, with 21% of HCV + PWID treated annually (among PWID engaged in harm reduction settings) (Appendix, Section 4).

**TABLE 2:** Model outcomes, by scenario, 2015, 2019 and 2030

Outcome	Scenario	Annual cases				WHO Target
		2015	2019	2030	Percent change^a^	
Viremic infections (total population)	Base	40 600	29 700	6500	−84%	—
	WHO Targets	40 600	29 700	4900	−88%	
Viremic infections (PWID)	Base	11 200	9700	7700	−31%	—
	WHO Targets	11 200	9700	70	−99%	
Incident infections (PWID)	Base	1300	1200	1200	−8%	−90%
	WHO Targets	1300	1200	120	−91%	
Liver‐related deaths	Base	170	100	20	−88%	−65%
	WHO Targets	170	100	20	−88%	

Abbreviations: PWID, people who inject drugs; WHO, World Health Organization.

^a^ Percent (%) change was calculated as the 2030 value divided by the 2015 value minus one.

## 4 DISCUSSION

Before the COVID‐19 pandemic introduced disruptions to hepatitis testing and care, Sweden was already experiencing a decline in the number of patients initiating treatment annually. Although Sweden was on‐track to meet most of the WHO targets by 2030, a 90% reduction in incidence could not be achieved. Using a dynamic transmission model based on the inputs described in Appendix, Section 1, our study calculated an annual HCV incidence rate of 29/100 PWID, among PWID sharing injecting equipment. This is in line with a recent study in NSP participants, which found an annual incidence rate of 22/100 person years (26/100 among HCV naïve and 19/100 among the spontaneously cleared group).^13^ Temporary declines in HCV treatment due to COVID‐19 were not found to impact long‐term progress towards these targets (i.e. the targets Sweden was projected to achieve under the 2019 base scenario would still be achieved, and targets Sweden was not projected to achieve under the 2019 base scenario would still not be achieved). However, excess morbidity and mortality would be expected, with increasing burden associated with longer disruptions to care.

A perhaps greater concern is that although more than 80% of HCV+ patients in Sweden currently are estimated to have been diagnosed, most infectious diseases clinics no longer have patients waiting for treatment. As a result, the number of patients treated annually is declining. The proportion of people previously diagnosed is based on cases notified to the PHA and previous publications^1^; however, this estimate has not been assessed by any recent general population studies. One study among pregnant women and their partners found that more than 83% of persons testing positive for HCV‐RNA had been previously diagnosed, and more than 50% of the diagnosed persons were previously lost to follow up.^14^ In order to eliminate HCV, all previously diagnosed patients need to be rescreened or reidentified and linked to care—either in a traditional setting or in a setting in which they are already linked to care (e.g. prisons, NSP, addiction clinics and treatment homes). During the COVID‐19 pandemic, screening activities for HCV seem to have decreased across settings, including primary care, with fewer referrals to HCV care centres in hospital settings as well as delays in scheduling among patients referred to infectious disease clinics (personal communication with the authors). This is likely due to a combination of factors, including limited physical visits by some healthcare facilities and also fewer visits by the patients for fear for COVID‐19. Also, the introduced initiatives of outreach programmes from departments of infectious diseases at the hospital settings may have reduced substantially due to the ongoing pandemic. All of this underscores the need for developing sustainability in treatment for HCV amidst COVID‐19. This includes increasing the access to treatment by outreach programmes through different settings, broadening provider base, and meeting patients where they are at.

To reduce HCV related mortality, continued screening for HCC with early detection of cancer in patients with liver cirrhosis is important. However, patient concerns about the safety of healthcare facilities due to COVID‐19 may prevent them from seeking care. Clear messaging is needed to ensure continuity of care, especially for patients who are most at risk. One limitation of our study is that HCV‐related mortality was calculated within the model, rather than evaluated using empirical data from Sweden, and is presented as the number of deaths among persons who are still HCV‐RNA+. However, a reduction in mortality is supported by the results of a recent systematic review that found SVR attainment to be a significant predictor of reduced mortality.^15^

Achieving the WHO targets can only be accomplished through the continued expansion of harm reduction services and treatment of high‐risk populations. Increased access to OST programmes is necessary; however, at least 50% of PWID in Sweden inject amphetamines and would not be reached by these efforts. As a result, NSP expansion in Sweden should be of primary importance to reach PWID in general and PWID who use amphetamine in particular.^16^ NSP programmes were first introduced in Sweden in 1986, but only began to scale up in 2017 following a change in law (more details in Appendix, Section 1). By the end of 2017, eight (of 21) regions offered NSP programmes, which increased to 16 regions by the end of 2019. However, although services may be available across most/all regions, that does not guarantee that PWID will access services at the same rate. Access to HCV treatment within harm reduction settings has improved substantially over the last few years and contributed to declining prevalence among participants, not only providing benefit to the individuals cured of their HCV infection but also reducing the likelihood of HCV transmission. However, increasing the number of avenues for accessing HCV treatment is sorely needed and can include increased providers in harm reduction settings, psychiatry settings, substance use disorder settings and prison settings.

A number of challenges remain, including identifying patients through new screening efforts or by tracking down patients previously lost to follow up. HCV is a notifiable disease, so all persons diagnosed with HCV are included in national registers. For the last few years, regions have been using these registers to try to reach patients who have been lost to follow‐up. However, this approach is complicated because some patients are hard to reach or may not be interested in further testing and treatment. This is amplified among legal minors who inject drugs as they are restricted by Swedish legislation from accessing NSP programmes. Further challenges may include convincing patients of the importance of treatment and/or prevention measures to avoid transmission or reinfection.

Underscoring all of this work is a need for sustainability in HCV outreach and care programmes that meet patients where they are. The traditional model of HCV treatment in infectious disease clinics and hospital settings is insufficient for HCV elimination, as has been exposed through the COVID‐19 pandemic. Expanded access to care should include education for all healthcare providers who interact with HCV positive persons, providing more access points for HCV diagnosis, treatment and care. Peer support through local drug user unions should be sought to alleviate loss to follow up and facilitate HCV treatment among PWID. And timely, ongoing, HCV screening with reliable linkage to treatment and care will be important for quickly identifying new infections and reinfections before patients are lost to follow up. Lastly, current regional reimbursement challenges may discourage smaller practices from treating for HCV, which should be addressed.

## 5 CONCLUSION

COVID‐19 has impacted HCV treatment as well as NSP and OST in Sweden; but the full extent of the disruptions is yet to be seen. As a result, it is of utmost importance that Sweden, and all countries, find sustainability in treatment by broadening the setting and base of providers to provide stability and continuity of care during turbulent times. Through increased harm reduction efforts and treatment among PWID, Sweden can reduce the incidence of HCV by 90% by 2030 and achieve the WHO targets. This will require a concerted effort and prioritization of hepatitis at a time when patients may be reluctant or unable to seek traditional care.

## CONFLICTS OF INTEREST

S.B. and D.R.‐S. are employees of the Center for Disease Analysis Foundation (CDAF). Over the past 3 years, CDAF has received research funding from Gilead, AbbVie and Vaccine Impact Modeling Consortium. CDAF has also received grants from CDC Foundation, John Martin Foundation, ASTHO, Zeshan Foundation and private donors. A.‐S.D. has received honoraria for lectures/consultancy from AbbVie, Gilead and MSD. A.J. has received research support from AbbVie and honoraria from MSD. M.K. has received honoraria for lectures from AbbVie, Gilead, MSD, Mundipharma, DnE Pharma and Nordic Drugs and has received research grants from Gilead and Nordic Drugs. P.‐E.K. has received honoraria for lectures from Gilead. H.R. has been a member of advisory boards for Gilead, AbbVie, Merck and VBI Vaccines. All proceeds are donated to CDAF. He is the managing director of Center for Disease Analysis (CDA) and CDAF. S.A. has received honoraria for lectures/consultancy from AbbVie, BMS, Gilead, MSD, and has received research grants from AbbVie and Gilead.

## Supporting information

Supplementary Material
