# Supplementary material for: Hepatitis C elimination in Sweden: Progress, challenges and opportunities for growth in the time of COVID‐19
Source: Liver Int. 2021 Jun 30;41(9):2024–31. doi: 10.1111/liv.14978 (PMC8242794; doi:10.1111/liv.14978)
Supplement: Supplementary file 1 — Supplementary Material [file LIV-41-2024-s001.docx]

**Appendix:**

**Hepatitis C Elimination in Sweden: Progress, Challenges and Opportunities for Growth in the time of COVID-19**

Blach S, Blomé M, Duberg A-S, Jerkeman A, Kåberg M, Klasa P-E, Lagging M, Razavi-Shearer D, Razavi H, Aleman S

**Contents**

[Section 1 2](#_Toc72139549)

[Modeling new infections 2](#_Toc72139550)

[Inputs Identified for Transmission Modeling 2](#_Toc72139551)

[History of NSP in Sweden 3](#_Toc72139552)

[Sensitivity analysis 3](#_Toc72139553)

[Section 2. Diagnosed and treated patients, 4](#_Toc72139554)

[Table 1. HCV newly diagnosed (Anti-HCV or HCV-RNA positive), by month, 2019 and 2020^15^ 4](#_Toc72139555)

[Table 2. HCV treatment initiations, InfCare Hepatitis, by month, 2019 and 2020^16^ 4](#_Toc72139556)

[Section 3. Scenario analysis: Impact of COVID delay scenarios and a scenario where treatment is fully stopped beginning in 2021 5](#_Toc72139557)

[Section 4. Scenario analysis: Impact of 90% NSP by 2023 under a variety of treatment scenarios on new viremic infections among PWID, 6](#_Toc72139558)

[References 7](#_Toc72139559)

# Section 1

## Modeling new infections

The model calculated new infections among PWID dynamically, considering the probability of infection, size of the susceptible population and proportion of the population sharing needles. Among PWID who did not share injecting equipment, the probability of infection was calculated considering only the background HCV prevalence in Sweden. For PWID who shared injecting equipment, the probability of infection was calculated using the average annual number of unsafe injections, HCV prevalence among shared syringes, and probability of HCV transmission from a contaminated needle (5%^1^). Participation in an NSP was assumed to result in a 30% reduction in unsafe injections, with OST participation resulting in a 50% reduction in unsafe injections. Simultaneous involvement in an NSP and OST was assumed to result in an 75% reduction in unsafe injections.^2-7^

## Inputs Identified for Transmission Modeling

In 2015, there were an estimated 21,000 people who inject drugs (PWID), in Sweden. This was estimated assuming 10,000 injecting opioid users and 11,000 injecting amphetamine users^8^ (Expert input based on Project Delta). In 2017, there were 4,468 clients on opiate substitution therapy (OST) (EMCDDA 2019 HSR-3). Data from 2013 suggest that 76.86% of people entering OST were still injecting (EMCDDA 2015 PDU-104); however, the proportion who continue to inject while on OST was not collected. Expert consensus suggests that an estimated 50% of OST participants continue to inject while on OST.

In 2019, there were at least 4,325 unique participants at Swedish needle and syringe programs (NSP) (InfCare NSP data, which represent >95% of all Swedish NSP participants, provided by Martin Kåberg). In the same year, the InfCare NSP quality register reported that more than 1,247,203 sterile needles were distributed, corresponding to 194-203 sterile syringes distributed per NSP participant per year. Data from program entrants into Stockholm NSP show that 29-34%^9^ had shared syringes or other paraphernalia within the last month; however, over time sharing of needle/syringes and other paraphernalia was reduced significantly to 14% and 24% respectively.^9^ Expert consensus suggests that 60% of all PWID shared syringes over the last year.

The HCV prevalence in OST populations is estimated to be around 50%, with a higher prevalence among NSP participants (closer to 60%). Overall, the group decided a 50% viremic prevalence for all PWID would be reasonable in 2018. The number of PWID initiating DAA treatment per year is uncertain. Treatment is available in OST centers, with some treatment also available onsite at a limited number of NSP. In addition, some PWID who are engaged with NSP or OST programs are treated off-site, where treatment is not available on-site.

## History of NSP in Sweden

Needle and syringe programs (NSP) were first introduced in Sweden in the mid 1980’s, as a WHO promoted response to HIV transmission among PWID. However, due to strict legislation, NSPs were only allowed by special permission from the Swedish board of Health and Welfare in the two cities of Lund and Malmö in Skåne County in southern Sweden. These two NSPs functioned as the only ones of their kind for more than two decades. They were continuously evaluated and the effect on HIV and HBV was indisputable, although the prevalence of HCV remained high during these first years.^10-13^ Legislation did not allow for the introduction of more NSPs until 2006, although the third Swedish NSP was not opened until 2010, also in Skåne County. Thus, there was a noticeable variation in regional NSP coverage. In 2013, a NSP was introduced in the capital city of Stockholm. In 2015, the UN pointed out that the restrictive drug policy in Sweden, including low access to NSPs was a violation against human rights. After this, the Public Health Agency released guidelines on HIV/hepatitis prevention among PWID further promoting the implementation of NSP nationally.^14^ Since 2017, NSP access has escalated rapidly - from 8 (of 21) regions offering programs by the end of 2017 to 16 regions in 2019.

## Sensitivity analysis

A one-way sensitivity analysis was conducted to measure the impact of the efficacy of harm reduction programs in reducing the incidence of HCV infections among PWID on the uncertainty in the reduction in incident cases of chronic HCV among PWID relative to 2015. For this, the respective efficacies of NSP, OST, and NSP & OST were assumed to be betaPERT-distributed with the likeliest, the lowest, and the highest values of the distribution obtained by setting the rates of infection (relative to that among the general PWID) to those shown in the table below.

| **Program** | **Probability of infection, relative to the general PWID population** |
| --- | --- |
| NSP | 0.70 (+/-20%) |
| OST | 0.50 (+/-20%) |
| NSP&OST | 0.25 (+/-20%) |

# Section 2. Diagnosed and treated patients,

## Table 1. HCV newly diagnosed (Anti-HCV or HCV-RNA positive), by month, 2019 and 2020^15^

|  | **Jan** | **Feb** | **Mar** | **Apr** | **May** | **Jun** | **Jul** | **Aug** | **Sep** | **Oct** | **Nov** | **Dec** |
| --- | --- | --- | --- | --- | --- | --- | --- | --- | --- | --- | --- | --- |
| **2019** | 122 | 118 | 127 | 112 | 135 | 104 | 116 | 104 | 119 | 144 | 104 | 90 |
| **2020** | 100 | 81 | 101 | 79 | 77 | 81 | 72 | 81 | 106 | 89 | 79* | 69* |
| **Change** | -18% | -31% | -20% | -29% | -43% | -22% | -38% | -22% | -11% | -38% | -24%* | -24%* |
| *The average change in diagnosed patients from August to October was assumed to remain constant through Nov and Dec | | | | | | | | | | | | |

## Table 2. HCV treatment initiations, InfCare Hepatitis, by month, 2019 and 2020^16^

|  | **Jan** | **Feb** | **Mar** | **Apr** | **May** | **Jun** | **Jul** | **Aug** | **Sep** | **Oct** |
| --- | --- | --- | --- | --- | --- | --- | --- | --- | --- | --- |
| **2019** | 598 | 527 | 446 | 396 | 391 | 292 | 192 | 213 | 391 | 446 |
| **2020** | 327 | 273 | 206 | 115 | 141 | 139 | 70 | 110 | 204 | 190 |
| **Change** | -45% | -48% | -54% | -71% | -64% | -52% | -64% | -48% | -48% | -57% |
| *At the time of the analysis, InfCare data were only available through October 2020 | | | | | | | | | | |

Section 3. Scenario analysis: Impact of COVID delay scenarios and a scenario where treatment is fully stopped beginning in 2021 on total viremic cases, liver related deaths and incident HCC; and the number of patients initiated treatment under each scenario, 2015-2030.

*****The scenario to discontinue treatment (zero patients treated after 2020) has not been described in-text, as it is highly unlikely to occur. However, it is provided here to show a “worst-case” scenario.

|  | **Total Initiating Treatment** | | | | | | | | |  |
| --- | --- | --- | --- | --- | --- | --- | --- | --- | --- | --- |
| **Scenario** | **2019** | **2020** | **2021** | **2022** | **2023** | **2024** | **2025** | **2026** | **2027+** | |
| 2019 Base | 4,800 | 3,200 | 3,200 | 3,200 | 2,500 | 2,500 | 2,500 | 2,500 | 2,500 | |
| 1-year | 4,800 | 2,100 | 3,200 | 3,200 | 2,500 | 2,500 | 2,500 | 2,500 | 2,500 | |
| 2-year | 4,800 | 2,100 | 2,100 | 3,200 | 2,500 | 2,500 | 2,500 | 2,500 | 2,500 | |
| 5-year | 4,800 | 2,100 | 2,100 | 2,100 | 2,100 | 2,100 | 2,500 | 2,500 | 2,500 | |
| Discontinue | 4,800 | 2,100 | 0 | 0 | 0 | 0 | 0 | 0 | 0 | |

Section 4. Scenario analysis: Impact of 90% NSP by 2023 under a variety of treatment scenarios on new viremic infections among PWID, compared with the 2019 Base and WHO Targets scenarios; and percent of total treatments, by population and harm reduction status under each scenario, 2015-2030.

**z**

# References

[1] Centers for Disease Control and Prevention. Recommendations for follow-up of health care workers after occupational exposure to hepatitis C virus. MMWR. 1997;46:603-6.

[2] Tsui JI, Evans JL, Lum PJ, Hahn JA, Page K. Association of opioid agonist therapy with lower incidence of hepatitis C virus infection in young adult injection drug users. JAMA internal medicine. 2014;174(12):1974-81.

[3] Turner KM, Hutchinson S, Vickerman P, Hope V, Craine N, Palmateer N, et al. The impact of needle and syringe provision and opiate substitution therapy on the incidence of hepatitis C virus in injecting drug users: pooling of UK evidence. Addiction. 2011;106(11):1978-88.

[4] Van Den Berg C, Smit C, Van Brussel G, Coutinho R, Prins M. Full participation in harm reduction programmes is associated with decreased risk for human immunodeficiency virus and hepatitis C virus: evidence from the Amsterdam Cohort Studies among drug users. Addiction. 2007;102(9):1454-62.

[5] White B, Dore GJ, Lloyd AR, Rawlinson WD, Maher L. Opioid substitution therapy protects against hepatitis C virus acquisition in people who inject drugs: the HITS-c study. Med J Aust. 2014;201(6):326-9.

[6] Hagan H, Pouget ER, Des Jarlais DC. A systematic review and meta-analysis of interventions to prevent hepatitis C virus infection in people who inject drugs. J Infect Dis. 2011;204(1):74-83.

[7] MacArthur GJ, van Velzen E, Palmateer N, Kimber J, Pharris A, Hope V, et al. Interventions to prevent HIV and Hepatitis C in people who inject drugs: a review of reviews to assess evidence of effectiveness. Int J Drug Policy. 2014;25(1):34-52.

[8] Jerkeman A. Hepatitis C virus infection in patients receiving opiate substitution therapy in Sweden. Lund University Faculty of Medicine Doctoral Dissertation Series: Lund University; 2014.

[9] Kåberg M, Karlsson N, Discacciati A, Widgren K, Weiland O, Ekström AM, et al. Significant decrease in injection risk behaviours among participants in a needle exchange programme. Infect Dis (Lond). 2020;52(5):336-46.

[10] Des Jarlais DC, Friedman SR, Choopanya K, Vanichseni S, Ward TP. International epidemiology of HIV and AIDS among injecting drug users. AIDS. 1992;6(10):1053-68.

[11] Månsson AS, Moestrup T, Nordenfelt E, Widell A. Continued transmission of hepatitis B and C viruses, but no transmission of human immunodeficiency virus among intravenous drug users participating in a syringe/needle exchange program. Scand J Infect Dis. 2000;32(3):253-8.

[12] Blome MA, Bjorkman P, Flamholc L, Jacobsson H, Molnegren V, Widell A. Minimal transmission of HIV despite persistently high transmission of hepatitis C virus in a Swedish needle exchange program. J Viral Hepat. 2011;18(12):831-9.

[13] Alanko Blome M, Bjorkman P, Flamholc L, Jacobsson H, Widell A. Vaccination against hepatitis B virus among people who inject drugs - A 20year experience from a Swedish needle exchange program. Vaccine. 2017;35(1):84-90.

[14] Karlsson N, Berglund T, Ekström AM, Hammarberg A, Tammi T. Could 30 years of political controversy on needle exchange programmes in Sweden contribute to scaling-up harm reduction services in the world? Nordic Studies on Alcohol and Drugs. 2021;38(1):66-88.

[15] Folkhälsomyndigheten [The Public Health Agency of Sweden]. Data & Statistics: Hepatitis C, 1999-2020. : Folkhälsomyndigheten; 2020 [Available from: <https://www.folkhalsomyndigheten.se/folkhalsorapportering-statistik/statistikdatabaser-och-visualisering/sjukdomsstatistik/hepatit-c/>.

[16] Nationella Kvalitetsregister. Annual Report InfCareHepatitis 2019. 2020.
